# Activation of SIRT1 by Hydroxysafflor Yellow A Attenuates Chronic Unpredictable Mild Stress‐Induced Microglia Activation and Iron Death in Depressed Rats

**DOI:** 10.1002/brb3.70385

**Published:** 2025-03-09

**Authors:** Jianle He, Min He, Ping Yang, Jianhui Shangguan, Lingxia Jiang, Zhiqiang Liu

**Affiliations:** ^1^ The Second Department of Neurology Jiangxi Provincial People's Hospital, The First Affiliated Hospital of Nanchang Medical College Nanchang Jiangxi China

**Keywords:** depression, ferroptosis, hippocampus, hydroxysafflor yellow A, microglia activation

## Abstract

**Background:**

Hydroxysafflor yellow A (HSYA), the main active ingredient in safflower, possesses antioxidant and anti‐inflammatory activities. We confirmed in our previous study that HSYA exerts antidepressant effects, but further investigation is needed to uncover the exact mechanism. Herein, we aimed to explore the antidepressant effects of HSYA based on microglial activation and ferroptosis studies.

**Methods:**

The chronic unpredictable mild stress (CUMS) procedure was used to establish a depression model in rats. Behavioral tests were conducted on rats after HSYA administration. Iba‐1 immunostaining was used to determine the activation of microglia in the hippocampus. We examined the iron ion level using a colorimetric method. Assayed by western blot for protein expression.

**Results:**

Rats receiving HSYA showed enhanced spatial learning and memory abilities, as well as improvements in depression‐like behaviors. HSYA administration reduced Iba‐1 expression in CUMS rats’ hippocampus, indicating that HSYA suppressed microglial activation. HSYA inhibited CUMS‐induced Fe^2+^ concentration and promoted ferroptosis‐related protein GPX4 and SLC7A11 expression. HSYA treatment also elevated SIRT1 and Nrf2 protein levels, while p‐p65 protein levels decreased in the hippocampus of CUMS rats.

**Conclusion:**

HSYA exerts an antidepressant‐like effect by inhibiting microglia activation in the hippocampus and inducing SIRT1/Nrf2/NF‐kB signaling.

## Graphical abstract

Schematic diagram illustrating the function of HSYA in CUMS‐induced rats. HSYA administration alleviated the depressive behaviors of CUMS‐induced rats and inhibited microglial activation and ferroptosis in the hippocampus through the activation of the SIRT1/Nrf2/NF‐kB pathway.

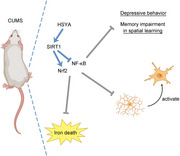

## 1 Introduction

Depression is a prevalent mental health condition that impacts mood, cognition, and actions (Goodwin and Stein 2021). Approximately 5% of adults in the world are diagnosed with depression each year, which has significant consequences for both individuals and society, such as increased suicide risk, higher cardiovascular disease risk, diminished social functioning, and economic repercussions (Shorey et al. 2022; Liao et al. 2023). In China, depression is ranked second among causes of disability, further emphasizing its importance as a public health issue (X. Li, Tian, et al. 2023; P. Chen, Bai, et al. 2022). The treatment of depression faces numerous obstacles, including low therapy rates, a slower onset of treatment, and comorbidities (Alnefeesi et al. 2022). Therefore, identifying new antidepressant drugs is of great importance.

Recently, traditional Chinese medicine (TCM) has attracted more attention due to its advantages of multi‐targets, multi‐approaches, whole regulation, and fewer adverse reactions (Ruan et al. 2023; Zhuang et al. 2023). Hydroxysafflor yellow A (HSYA), derived from safflower, has shown promising biological activity in various diseases (Xue et al. 2021). HSYA, for instance, demonstrated neuroprotective properties in acute traumatic brain injury and mitigated dopaminergic neurodegeneration in Parkinson's disease models through antioxidant and anti‐inflammatory mechanisms (Lai et al. 2023; Yang et al. 2020). Moreover, HSYA effectively attenuates atherosclerotic plaque formation in diabetic mice with atherosclerosis and inhibits iron‐induced endothelial cell apoptosis (Rong et al. 2023). We have previously demonstrated that HSYA exerts antidepressant effects (Liu et al. 2022), but the specific mechanism of action still needs to be studied.

This study used chronic unpredictable mild stress (CUMS) to establish a rat model of depression, further clarifying the antidepressant effects and underlying mechanisms of HSYA.

## 2 Materials and Methods

### 2.1 Establishment of the Rat Depression Model via Chronic Unpredictable Mild Stress (CUMS)

A total of 30 Sprague–Dawley male rats (100～120 g) were obtained from Beijing Vital River Laboratory.

An adaptive feeding protocol was conducted for 1 week under standard conditions (temperature of 21°C～23°C, humidity of 50%～60%, and 12 h lighting and dark periods). A microisolator cage provided free access to food and water for rats during the experiments.

The CUMS procedure was employed to induce a depression model in rats (Willner 2017). Briefly, during a 4‐week period, rats were raised individually in polycarbonate cages and exposed to daily stress regimes. A total of 10 stressors were included: food deprivation 24 h, water deprivation 24 h, wet bedding 24 h, alcohol odor stimulus 10 min, reversed light/dark cycle 24 h, physical restraint 2 h, light stimulation during the night 24 h, tail clamping 3 min, no bedding 24 h, and cage shaking 2 h. A random order of two stressors was administered to rats every day. Rats in the control group were fed standard diets.

CUMS rats were randomly classified into four groups (six per group): CUMS, CUMS+HSYA (15 mg/kg), CUMS+HSYA (30 mg/kg), and CUMS+HSYA (60 mg/kg). After exposure to CUMS, each group of rats was intragastrically administered the corresponding concentration of HSYA (Cas#: 78281‐02‐4, purity 98%, the Chinese Academy of Food and Drug Control, Beijing, China) once a day for 21 days, using a solution of HSYA dissolved in 0.9% NaCl saline. Behavioral tests were then conducted. All animal experiments were conducted by randomization with blinded investigators. All animal experiments were approved by the Ethics Committee of Jiangxi Provincial People's Hospital (Ethics number: 2023‐59) and conducted according to the National Institutes of Health Laboratory Animal Care and Use Guidelines.

### 2.2 Y Maze Test

Rats were tested on their spatial recognition memory using the Y Maze. There were three identical arms in the maze: the starting arm A, the new arm B, and the other arm C. As part of the training phase, a partition blocked access to the new arm B, allowing rats to explore the starting arm A and other arms C for 10 min. After training, rats were placed in 1 h retention intervals. Subsequently, the partition blocking the new arms B was removed, and rats were free to navigate the Y maze (A, B, and C) for 8 min during the detection phase. Rat behavior was documented during an 8‐min period through video recordings. A percentage of time rats spent in the new arm B was calculated.

### 2.3 Elevated Plus Maze Test (EPM)

Exploratory and anxiety‐related behaviors in rats were evaluated by EPM. This maze comprises a central platform and four arms extending from it, with the platform positioned at a height of 80 cm. Two of these arms are enclosed by high walls, designated as closed arms, while the remaining two arms are open. Rats were positioned on the central platform facing the open arm and permitted to freely explore the maze for 5 min, during which their time spent on the open arms was documented via video recording.

### 2.4 Novelty‐Suppressed Feeding Test (NSFT)

For NSFT, rats were deprived of food for 24 h. A new chamber containing a central platform with unfamiliar food was then prepared. We placed rats in the corner of the chamber facing away from the new food. The latency feeding time was recorded, which is the time from rats’ initial entry into the chamber until they began consuming the new food. Typically, a shorter incubation period indicates reduced anxiety and depression.

### 2.5 Immunofluorescence (IF) Staining

Pentobarbital (50 mg/kg) was injected intraperitoneally into the rats after the behavioral tests above. Rats then received a transcardial perfusion of ice‐cold saline, followed by 4% paraformaldehyde. Each rat's brain was separated by skull stripping. Hippocampal tissue was quickly obtained after separating the cerebral cortex with an ophthalmic tweezer.

For IF staining, coronal sections (40 µm) were prepared using a vibratome (VT1200S, Leica Microsystems, Watzlar, Germany) after the brains were fixed in 4% paraformaldehyde for 24 h. Subsequently, 0.4% Triton X‐100 permeabilization was conducted, followed by blocking with 10% goat serum for 1 h. Sections were incubated overnight with anti‐Iba‐1 antibodies (ab178847, 1:200, Abcam) at 4°C, followed by a 2 h incubation with FITC‐labeled secondary antibodies. For nuclear visualization, DAPI staining was used. A Nikon E800 fluorescence microscope (Nikon, Tokyo, Japan) was used to examine and capture images of the hippocampal CA3 area.

### 2.6 Iron Content Measurement

Ferrous iron (Fe^2+^) levels in hippocampus tissue grinding fluid were measured using an Iron Assay Kit with colorimetric method (Abcam, Cambridge, UK) at 593 nm wavelength.

### 2.7 Western Blot

Hippocampal tissue lysate was prepared using RIPA lysis buffer (Beyotime, Shanghai, China), and the supernatant was obtained after boiling as protein samples. Protein samples underwent SDS‐PAGE and were transferred to a PVDF membrane. Following incubation with primary antibodies listed in Table 1 overnight at 4°C, peroxidase‐labeled secondary antibodies were applied for 2 h. After visualizing with the ECL solution (Beyotime), quantitation of the bands was done using ImageJ software.

**TABLE 1:** Antibodies used for western blot.

Antigen	Code	Working dilution	Supplier
Iba‐1	ab5076	1:1000	Abcam
GPX4	ab125066	1:1000	Abcam
SLC7A11	ab307601	1:1000	Abcam
SIRT1	ab110304	1:1000	Abcam
Nrf2	ab313825	1:1000	Abcam
p65	ab16502	1:1000	Abcam
p‐p65	ab76302	1:500	Abcam

### 2.8 Statistical Analysis

Data analysis was performed using GraphPad Prism 8.0 for a one‐way ANOVA followed by a Tukey post hoc test. Data was presented as mean ± SD. *p *< 0.05 indicates statistically significant differences.

## 3 Results

### 3.1 HSYA Ameliorates the Depressive Behaviors and Spatial Learning and Memory Disabilities of Rats Exposed to CUMS

**FIGURE 1:**
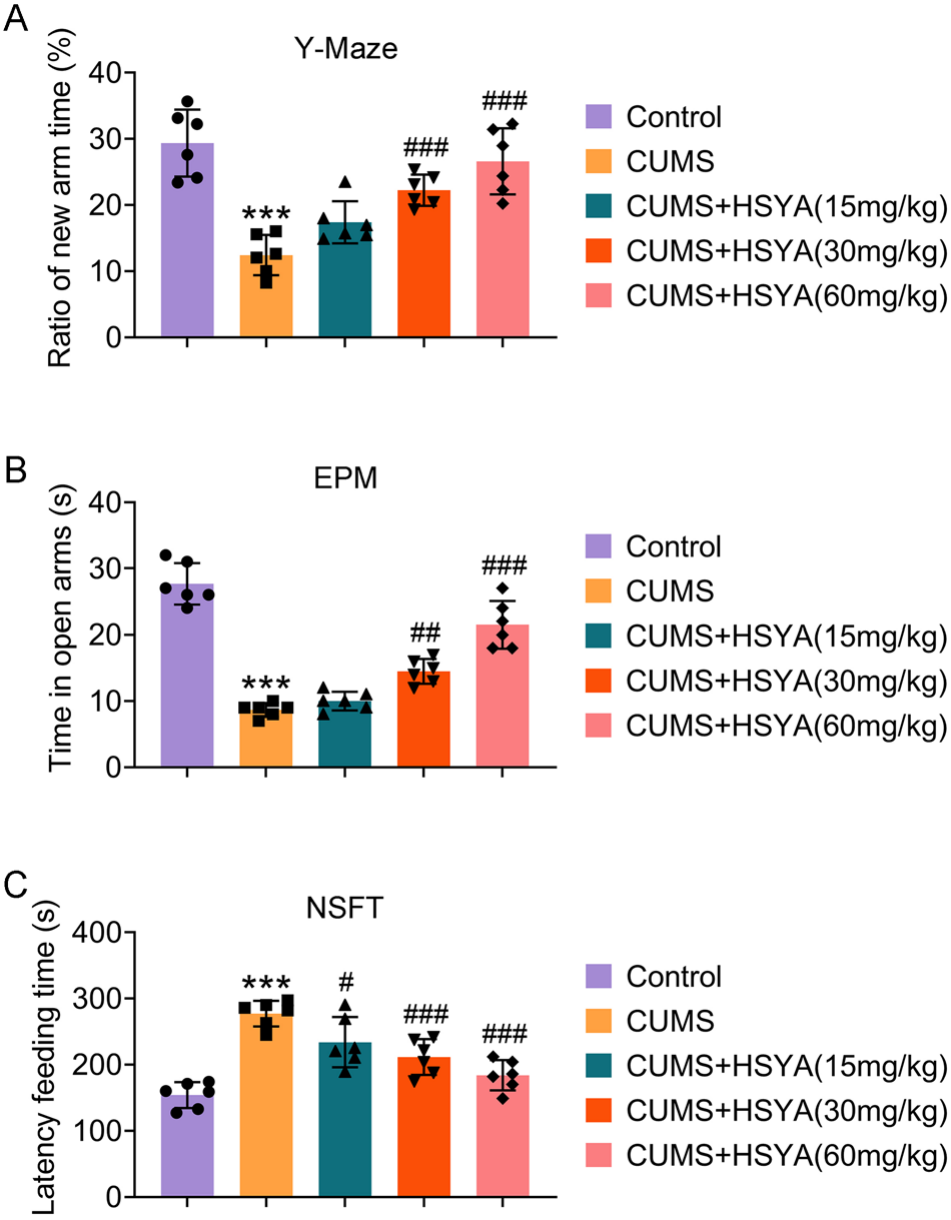
HSYA improves the depressive behaviors and spatial learning and memory impairment of CUMS rats. (A) The ratio (%) of time spent in the new arm time in the Y‐maze test. (B) Time spent in the open arms of EPM. (C) Latency time for NSFT feeding. ^***^ *p *< 0.001 versus control group; ^#^ *p *< 0.05, ^##^ *p *< 0.01 and ^###^ *p *< 0.001 versus CUMS group. For *p* values, *F* values, and degrees of freedom, see Table S1.

We first tested the spatial learning and memory abilities of each group using a Y maze test. The new arm visitation time in CUMS rats was reduced, while it was prolonged after HSYA administration (Figure 1A). CUMS rats showed a decrease in open arms and an increase in latency feeding time compared to the control group, according to EPM and NSFT. In contrast to CUMS, HSYA administration improved these symptoms (Figure 1BC). Based on these results, HSYA showed positive effects on cognitive functions and depression‐like behaviors in CUMS rats.

### 3.2 HSYA Inhibits Microglial Activation in the Hippocampus of CUMS Rats

**FIGURE 2:**
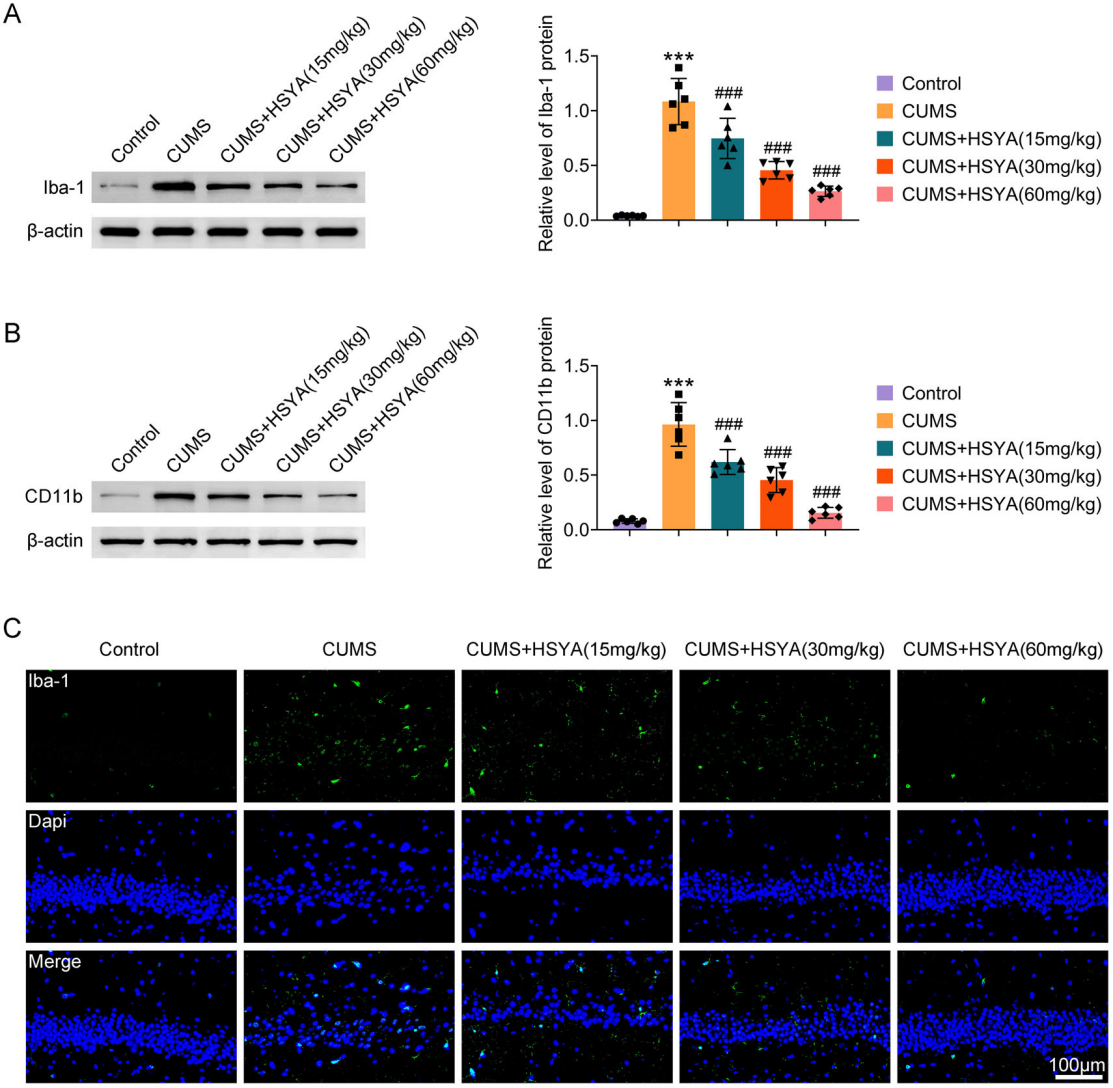
HSYA inhibits microglial activation in the hippocampus caused by CUMS. (A) Iba‐1 expression in the hippocampus was measured by western blot. (B) CD11b expression in the hippocampus was measured by western blot. (C) Iba‐1 expression in the hippocampus was examined using IF staining. ^***^ *p *< 0.001 versus control group; ^###^ *p *< 0.001 versus CUMS group. For *p* values, *F* values, and degrees of freedom, see Table S1.

Western blot analysis revealed that Iba‐1 and CD11b expressions were upregulated in the hippocampus of CUMS rats, which were greatly reversed by HSYA treatment (Figure 2AB). Further, IF staining of Iba‐1 in the hippocampus showed the same results (Figure 2C). Thus, HSYA treatment in CUMS rats reduces microglial activation in the hippocampus.

### 3.3 HSYA Mitigates Ferroptosis in the Hippocampus of CUMS Rats

**FIGURE 3:**
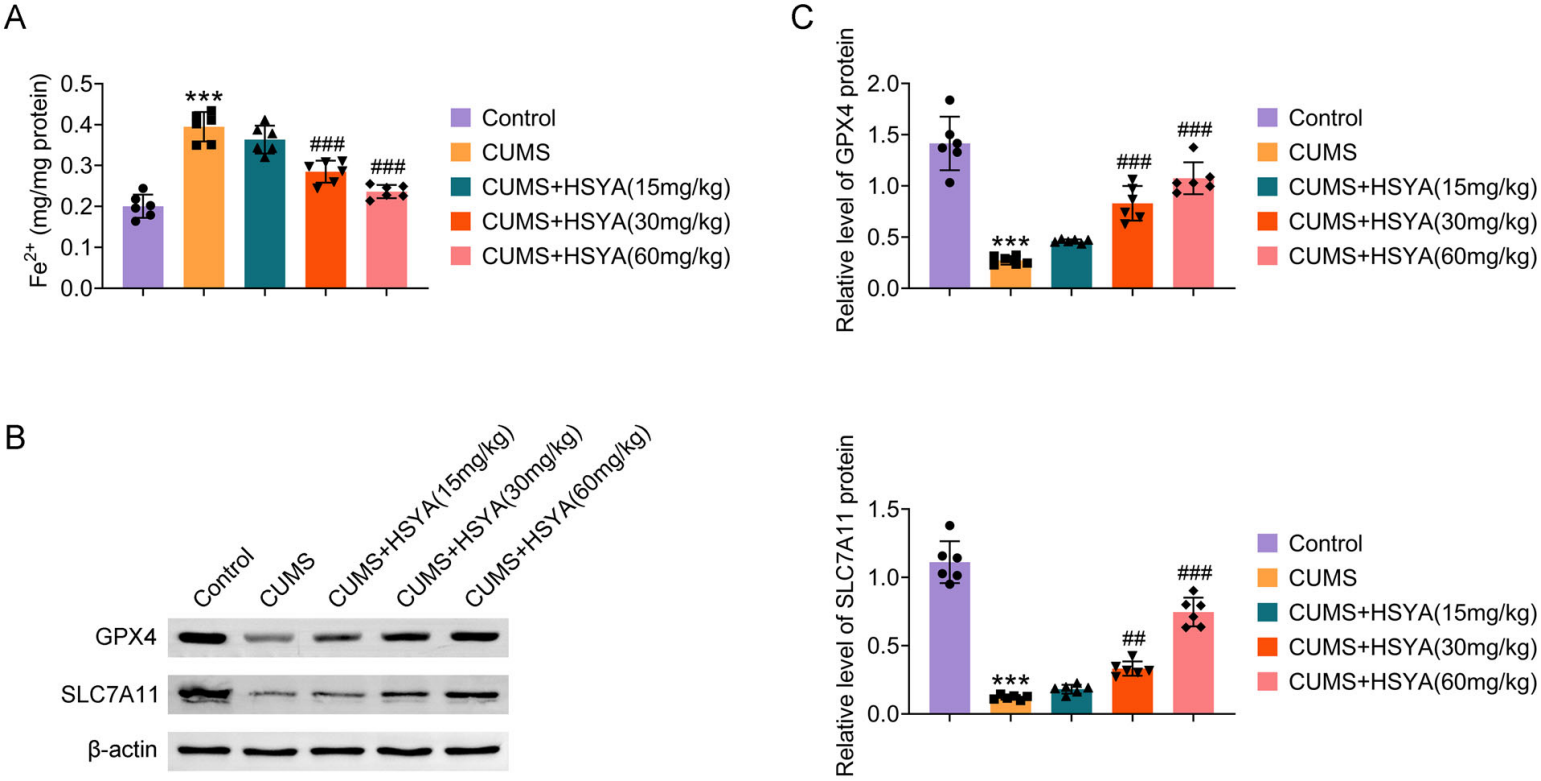
HSYA inhibits hippocampal ferroptosis in CUMS rats. (A) Fe^2+^ levels in the rat hippocampus were determined. (B) Ferroptosis‐related protein (GPX4 and SLC7A11) levels were measured by western blot. ^***^ *p *< 0.001 versus control group; ^##^ *p *< 0.01 and ^###^ *p *< 0.001 versus CUMS group. For *p* values, *F* values, and degrees of freedom, see Table S1.

The effect of HSYA on ferroptosis‐related markers, including Fe^2+^, GPX4, and SLC7A11, was examined in the hippocampus. Fe^2+^ concentration was elevated in the hippocampus of CUMS rats, while HSYA administration reduced Fe^2+^ concentration. (Figure 3A) Western blot of the hippocampus revealed downregulation of glutathione peroxidase 4 (GPX4) and recombinant solute carrier Family 7, Member 11 (SLC7A11) protein expression in CUMS rats, whereas HSYA administration increased their expression levels (Figure 3BC). These data suggest that HSYA inhibits ferroptosis in the hippocampus caused by CUMS.

### 3.4 HSYA Regulates SIRT1/Nrf2/NF‐kB Signaling Pathway Expression

**FIGURE 4:**
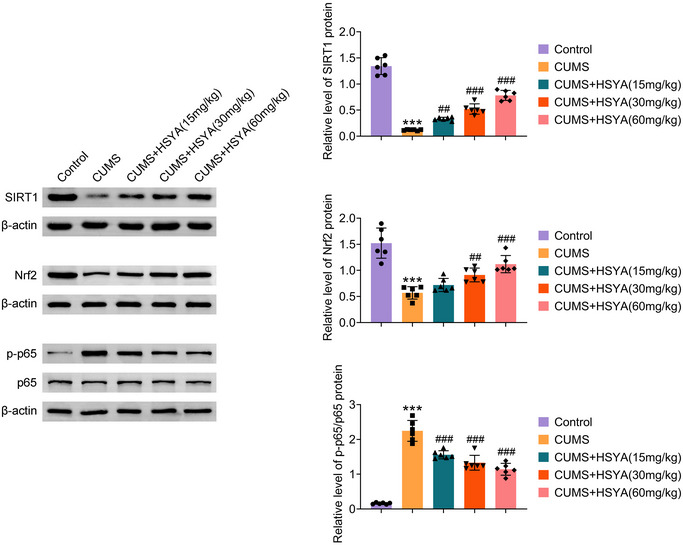
Effects of HSYA on SIRT1/Nrf2/NF‐kB signaling pathway. Western blot analysis was performed to assess SIRT1, Nrf2, p‐p65, and p65 protein levels. ^***^ *p *< 0.001 versus control group; ^##^ *p *< 0.01 and ^###^ *p *< 0.001 versus CUMS group. For *p* values, *F* values, and degrees of freedom, see Table S1.

The SIRT1/Nrf2/NF‐kB pathway participate not only in anti‐inflammatory and antioxidant processes, but also as neuropretective (Alqrad et al. 2023; Jin et al. 2021; N. Chen, Wang, et al. 2022). Further evaluation of HSYA's effects on the SIRT1/Nrf2/NF‐kB pathway was performed using western blot. Decreased levels of SIRT1 and Nrf2 proteins and an increased level of p‐P65 protein were observed in CUMS rats’ hippocampus (Figure 4). HSYA administration, however, significantly reversed these alterations. Based on the results, HSYA activates the SIRT1/Nrf2/NF‐kB pathway in the hippocampus of CUMS rats.

## 4 Discussion

Depression symptoms include loss of energy, thinking retardation, memory loss, and slow responses (Bai et al. 2023). Research on depression in animals has often used the CUMS model, which involves exposing rats to various unpredictable stressors to simulate real‐life stress scenarios encountered by humans, resulting in behavioral and neurobiological changes (Markov and Novosadova 2022; Strekalova et al. 2022; Nollet 2021). This study indicated that CUMS rats were treated with HSYA for 4 weeks, leading to improvements in spatial memory, learning ability, and depression‐like behaviors. HSYA's antidepressive properties are further supported by these results. Microglia are key immune cells within the central nervous system (G. Zhao and Jing 2023). Emerging research suggests that microglia sense depression‐related stressors and initiate immune responses and neuroinflammation that may contribute to depression pathogenesis (H. Wang et al. 2022; B. Li et al. 2022; Y. Sun, Zhao, et al. 2023). Microglial activation is confirmed to correlate with hypothalamic‐pituitary‐adrenal (HPA) axis activation (Cheiran Pereira et al. 2022). Persistent HPA axis activation chronically elevates glucocorticoids in depression. This leads to hippocampal atrophy, impairing emotional regulation and memory function, which are typical symptoms of depression (Keller et al. 2017). Glucocorticoids have anti‐inflammatory effects and inhibit microglial activation. However, in depression, persistent activation of the HPA axis decreases glucocorticoid receptor sensitivity, compromising the regulation of microglial activity and exacerbating depression symptoms (Cheiran Pereira et al. 2022; Picard et al. 2021). This study revealed that CUMS led to elevated Iba‐1 expression in rat hippocampal tissue, and treatment with HSYA counteracted this effect, indicating its potential to mitigate CUMS‐induced microglia activation. Notably, our previous studies demonstrated that HSYA reduces hippocampal inflammation and oxidative stress by modulating the HPA axis (Liu et al. 2022). This study contributes to a deeper understanding of the mechanisms by which HSYA exerts its anti‐inflammatory effects in the hippocampus via the HPA axis.

In ferroptosis, iron‐dependent programmed cell death, reactive oxygen species (ROS) are generated by iron ions oxidizing lipids and decreasing intracellular GPX4 levels (Yao et al. 2023; Jiang et al. 2021). Several studies have demonstrated iron deposition in depression animal models, which is related to depression severity (E. Li, Yin, et al. 2023; Jiao et al. 2021). This correlation is primarily attributed to an increase in oxidative stress, disruption of neurotransmitter synthesis, and impairment of synaptic plasticity (L. Wang et al. 2023). Consequently, addressing iron overload may enhance antidepressant efficacy. Prior research indicates that HSYA can mitigate hypoxic‐reperfusion‐induced neuronal and myocardial injuries by inhibiting ferroptosis (G. Chen. Li, et al. 2022; Qin et al. 2020). HSYA treatment reversed elevated levels of divalent iron ions and reduced levels of GPX4 and SLC7A11 that were found in the hippocampus of CUMS rats. HSYA suppresses ferroptosis in hippocampal cells, contributing to a better understanding of how it alleviates depression.

SIRT1, a NAD+‐dependent deacetylase, regulates various biological functions, including inflammation, apoptosis, and gene expression (Shen et al. 2021). Researchers previously showed that HSYA activated SIRT1 in microglia to mitigate LPS‐induced inflammation (Qin et al. 2020). SIRT1 has also been shown to modulate downstream targets such as Nrf2 and NF‐kB, and the SIRT1/Nrf2/NF‐kB pathway has been identified as a potential target for anti‐inflammatory, antioxidant, and ferroptosis inhibition effects in many drugs (Jin et al. 2021; N. Chen, Wang, et al. 2022; L. Sun, He, et al. 2023; L. Zhao et al. 2023). His study also demonstrated that HSYA activates SIRT1 and Nrf2 as well as inhibits p‐p65 expression in the hippocampus of CUMS rats, suggesting that HSYA may also impact depression by activating the SIRT1/Nrf2/NF‐kB pathway. However, further verification of this mechanism is needed.

In conclusion, HSYA is effective in treating depression. The underlying mechanism may include inhibition of microglia activation and ferroptosis in the hippocampus, as well as SIRT1/Nrf2/NF‐kB pathway activation.

## Author Contributions

**Jianle He**: methodology, conceptualization, data curation, formal analysis, writing–review and editing. **Min He**: conceptualization, methodology, formal analysis, data curation, writing–review and editing. **Ping Yang**: data curation, investigation. **Jianhui Shangguan**: validation, data curation. **Lingxia Jiang**: software, investigation. **Zhiqiang Liu**: conceptualization, methodology, data curation, formal analysis, validation, writing–original draft.

## Ethics Statement

Ethical approval was obtained from the Ethics Committee of Jiangxi Provincial People's Hospital (Ethics number: 2023‐59).

## Conflicts of Interest

The authors declare no conflicts of interest.

### Peer Review

The peer review history for this article is available at https://publons.com/publon/10.1002/brb3.70385.

## Supporting information



Supporting Information

## Data Availability

All data generated or analyzed during this study are included in this published article. The datasets used and/or analyzed during the present study are available from the corresponding author on reasonable request.
